# Provision of physical activity advice for patients with chronic diseases in Shenzhen, China

**DOI:** 10.1186/s12889-021-12185-7

**Published:** 2021-11-23

**Authors:** Rui Hu, Stanley Sai-chuen Hui, Eric Kam-pui Lee, Mark Stoutenberg, Samuel Yeung-shan Wong, Yi-jian Yang

**Affiliations:** 1 Department of Sports Science and Physical Education, The Chinese University of Hong Kong, Shatin, N.T Hong Kong; 2 School of Public Health and Primary Care, The Chinese University of Hong Kong, Shatin, N.T Hong Kong; 3 Department of Kinesiology, College of Public Health, Temple University, Philadelphia, PA USA

**Keywords:** Exercise is medicine, Chronic disease, Physician

## Abstract

**Background:**

Physical activity (PA) may best be promoted to patients during clinical consultations. Few studies investigated the practice of PA advice given by physicians, especially in China. This study aimed to investigate the prevalence and contents of PA advice given by physicians in China and its association with patients’ characteristics.

**Methods:**

Face-to-face questionnaire asking the prevalence and contents of PA advice given by physicians was administered to adult patients in three major hospitals in Shenzhen, China. Attitude of compliance, stature, PA level, and socio-demographic information were also collected. Data was analyzed via descriptive statistics and binary logistic regression.

**Results:**

Of the 454 eligible patients (Age: 47.0 ± 14.4 years), only 19.2% (*n* = 87) reported receiving PA advice, whereas 21.8%, 23.0%, 32.2%, and 55.2% of patients received advices on PA frequency, duration, intensity, and type, respectively. Male patients were more likely to receive PA advice from physicians [odds ratio (OR): 1.81; 95% confidence interval (CI): 1.08–3.05], whereas patients who were unemployed (OR: 0.16; 95% CI: 0.04–0.67), and who already achieved adequate amount of PA (OR: 0.29; 95% CI: 0.12–0.71) were less likely to receive PA advice.

**Conclusions:**

Prevalence of physicians providing physical activity advice to patients is low, there is a pressing need to take intervention measures to educate healthcare providers.

**Supplementary Information:**

The online version contains supplementary material available at 10.1186/s12889-021-12185-7.

## Background

Chronic diseases are the leading causes of death worldwide [1]. In 1990, > 28 million global deaths were due to chronic disease [2], which increased to 39.5 million deaths in 2016 [3]. Furthermore, three quarters of all deaths attributed to chronic diseases occurred in low- and middle-income countries [4]. For instance, China, a middle-income country, chronic diseases accounted for 86.6% of mortality in 2015 [5]. This chronic disease pandemic imposes a great burden to the healthcare system and effective interventions are imperative to alleviate the current situation [6].

Physical activity (PA) reduces the incidence and complications of chronic diseases among adults and offers other multiple health benefits including the prevention and treatment of psychiatric diseases, neurological diseases, pulmonary diseases, diabetes, musculoskeletal disorders, and cancer [7, 8]. In contrast, physical inactivity is responsible for almost one sixth of the direct (medical) and indirect (non-medical) yearly costs for the management of chronic diseases [9]. Despite its health and economic benefits, participation in PA among Chinese adults remains low [10].

Physicians are influential sources of health information [11]. The Exercise is Medicine (EIM) initiative, developed by the American College of Sports Medicine (ACSM) and the American Medical Association (AMA), calls upon physicians to address the physical inactivity pandemic in patients with chronic diseases by assessing PA levels, providing PA counselling and referring patients to PA resources [12]. Numerous studies have evaluated PA promotion (i.e., prescription and referral) in primary health care setting, concluding that PA promotion delivered via healthcare providers in clinical settings is effective to increase patients’ PA levels and improve their health outcomes [13–15].

Although PA promotion in clinical settings is effective, only a small number of patients received PA advice from their healthcare providers in western countries. It was reported that only 18.0, 32.8, and 56.0% of patients received PA advice from healthcare providers in Australia, Germany, and United States (U.S.), respectively [16–18]. Reasons for not recommending PA to patients are often related to limited consultation time and insufficient education in EIM, competing priorities during clinical consultations, and perceiving a lack of motivation in patients [19]. Other studies examined patients’ characteristics associated with the receipt of PA advice. In Australia, patients with a lower physical and mental health-related quality of life score and/or with chronic diseases were more likely to receive PA advice [16]. Similarly, in U.S., patients who were male, non-married, lower-educated, Spanish-speaking, and with no chronic conditions were less likely to receive PA advice [20].

The prevalence of PA advice and its associated patients’ characteristics are understudied in Eastern countries like China. Furthermore, only a few studies investigated the contents of PA advice being provided to patients [16, 21, 22]. Specific contents of PA advice should contain exercise type, intensity, frequency and duration of activities. PA advice lacking specific contents may limit the health impact to patients [16]. Therefore, the present study aimed to: 1) investigate the prevalence of PA advice in patients with chronic diseases in China (primary outcome); 2) describe the content of PA advice that patients with chronic disease receive and examine whether such PA advice was adequate as defined by the current PA guideline (secondary outcome) [23]; and 3) identify patients’ factors that were associated with the receipt of PA advice (secondary outcome).

## Methods

### Study population

Adult patients with chronic diseases were recruited from the three largest general hospitals in Shenzhen, China to participate in an in-person survey. These hospitals were selected because of their high patient volume. Patients were interviewed immediately after their visit with the physician, outside the clinic consultation room, in a randomized order.

Patients that met the following inclusion criteria were included in the study: 1) ≥18 years of age, 2) presence of chronic disease (i.e diseases that identified by EIM) [24], 3) who were able to complete a face-to-face survey verbally. Exclusion criteria included: 1) patients with aneurysm, cardiac pacemaker, mobility limitations, human immunodeficiency virus (HIV), 2) pregnancy, or 3) patients who were hospitalized.

Patients with chronic disease were identified through the following series of questions: “Did you see a physician?” Those who answered “yes” were further asked, “What is your primary purpose for your visit to physicians?” Patients who self-reported having a chronic disease, including heart disease (heart failure), peripheral arterial disease, hypertension, pre-diabetes, type 2 diabetes, blood lipid disorders, osteoarthritis, osteoporosis, rheumatoid arthritis, low back pain, fibromyalgia, asthma, chronic obstructive pulmonary disorder (COPD), cancer (colorectal cancer, prostatic cancer and breast cancer), depression or anxiety, chronic kidney or liver disease, inflammatory bowel disease, Parkinson’s disease and Alzheimer’s disease were invited to participate in the study [24].

By assuming the prevalence of PA advice was 50% (which would require the largest sample size), with a precision/absolute error of 5%, and at type I error of 5%, a total of 385 patients were required as according to the Charan and Biswas formula [25] for sample size calculation: Sample size = [(Z_1-α/2_)^2^p(1-p)]/d^2^, in which Z_1-α/2_ is standard normal variate (1.96 were used), p is expected proportion of patients received PA advice (i.e., 50%), and d is absolute error/precision (i.e., 0.05).

Written informed consent was obtained for all participants. All participation was voluntary and no incentive was involved. Ethical approval for the study was granted by the Survey and Behavioral Research Ethics Committee, the Chinese University of Hong Kong (Reference number. SBRE-20-026). All methods were carried out in accordance with the Declaration of Helsinki.

### Development of the questionnaire

Data were collected between September 2020 and October 2020. A total of 454 patients completed the questionnaire. The questionnaire was developed based on the Anderson’s Behavioral Model of Health Services Use, items from the Behavioral Risk Factors Surveillance System (BRFSS) questionnaire and previously published surveys from Canada and Australia [16, 26–30]. Anderson’s Behavioral Model was initially developed to understand how and why individuals use health services. It has been used in several areas of healthcare utilization and in relation to different type of diseases, such as predicting receipt of PA advice [18, 31, 32]. The final questionnaire consisted of 25 questions including multiple choice, dichotomous and open-ended items that explored the patients: 1) medical diagnosis, 2) socio-demographic characteristics, 3) anthropometric measurements, 5) self-reported PA levels, 5) receipt of PA advice from their physicians, and 6) likelihood of patients follow PA advice from their physicians. The questionnaire can be found at Additional file 1.

### Measures

#### Physical activity advice

The presence of PA advice was detected by the following question: “Did the physician provide PA advice to you just now?” Those who answered “yes” were then asked about the details of the advice including its frequency (i.e., number of times per week), its intensity (i.e., low, moderate or high intensity), its duration (i.e., minutes per session) and its type (e.g., walking, Taichi, square dance … etc).

#### Likelihood of patients following PA advice from their physician

Patients who received PA advice from their physicians were asked “How likely is it that you will follow the PA advice given by your physician?” Response options included, “I will follow the advice”, “I will not follow their advice”, and “I don’t know”.

#### Anthropometric measurements

Height (cm) and weight (kg) were self-reported and used to calculate body mass index (BMI) [33]. Patients were classified as underweight (< 18.5 kg/m^2^), normal weight (18.5 to 22.9 kg/m^2^), overweight (23.0 to 24.9 kg/m^2^), and obese (≥25 kg/m^2^), according to latest relevant World Health Organization (WHO) guideline [34].

#### Current PA behavior

Patients were asked whether they participated in PA within the last 6 months. Patients who answered “yes” were then further queried about the frequency, intensity, duration, and type of their regular PA. Patients who reported having regular moderate to vigorous intensity PA (MVPA), they were further being prompted to provide the total number of minutes they engaged in MVPA. Patients were then categorized into (i) patients who achieved adequate PA and patients who did not, according to the latest WHO guideline (i.e., engaging in 150 min of moderate to vigorous PA per week) [23].

#### Socio-demographic information

Socio-demographic information collected from the patients included gender, age, employment status (employed/unemployed/retired), and educational attainment (less than primary education/primary education/secondary education/college or associated degree/bachelor degree or higher). Familiarity with the hospital team was estimated by the number of years as a patient in the corresponding hospital as previous work has found this measure to be associated with health services utilization [30, 31].

### Statistical analysis

Descriptive data were presented as mean ± standard deviation (SD) or as a percentage. To detect whether physicians’ PA advice was adequate according to the current global PA guideline, each item (frequency, intensity, duration, and type) was coded according to the WHO PA guideline for individuals with chronic conditions [23]. Advice that received all four points should be in accordance to the aerobic PA guideline [aerobic activity (*type*: 1 point), over the course of a week (*frequency*: 1 point), at a moderate or vigorous *intensity* (1 point), for at least 75 min (if vigorous intensity) or 150 min (if moderate intensity) (*time*: 1 point)] [35]. Logistic regression analyses (unadjusted and adjusted) were utilized to identify factors associated with the presence of PA advice. The following independent variables were included: gender, age, employment status, educational attainment, BMI, years as a patient, and whether the patient met PA guideline within the last 6 months. Age and years as a patient were dichotomized based on their means for regression analysis. Univariate models were first obtained for each independent factor. The final adjusted model included all independent factors with a *p*-value < 0.15. A forward logistic regression was then utilized to identify factors significantly associated with receiving PA advice from physicians. Data analyses were performed using SPSS 26.0. A *p*-value < 0.05 was considered as statistically significant in the final logistic regression model.

## Results

**Fig. 1:**
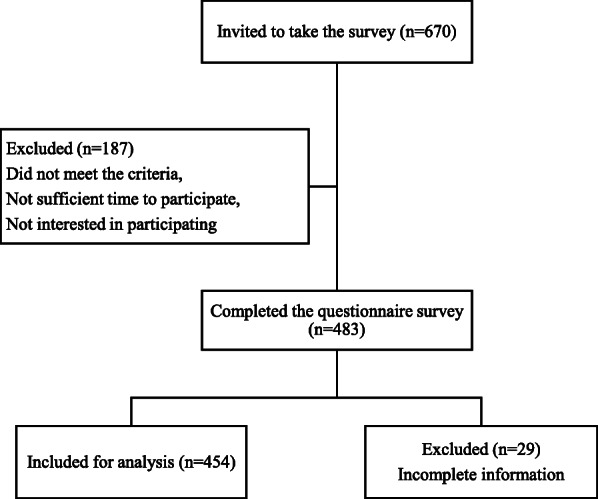
Flow chart outlining the response rate of the survey

Out of 670 patients contacted, 187 patients were excluded due to not meeting inclusion criteria, not have sufficient time for completing the questionnaire, or not interested in the survey (Fig. 1). Out of the 483 patients who completed the survey, 29 were further excluded for incomplete information, resulting in a final sample size of 454 for analysis (response rate = 67.8%).

The mean age of the respondents (*N* = 454) was 47.0 ± 14.4 years-old. A majority of respondents were women (51.3%, *n* = 229) and were employed (69.0%, *n* = 298). Those who completed post-secondary education accounted for 47.7% (*n* = 215) whereas secondary school 32.6% (*n* = 147). A total of 19.4% (*n* = 88) and 26.2% (*n* = 119) of the respondents were classified as overweight and obese, respectively. Furthermore, 18.3% (*n* = 76) of the respondents met PA guidelines within the last 6 months (Table 1).

**Table 1:** Demographic characteristics of study participants (*N* = 454)

Characteristics	n	Percentage/Mean (SD)
Gender		
Female	229	51.3%
Male	217	48.7%
Age, years		47.0 (14.4)
Education		
Less than secondary school	89	19.7%
Secondary school	147	32.6%
Post-secondary school	215	47.7%
Employment status		
Employed	298	69.0%
Unemployed	58	13.4%
Retired	76	17.6%
BMI, kg/m^2^		
Underweight	22	4.8%
Normal weight	225	49.6%
Overweight	88	19.4%
Obese	119	26.2%
No. of years as a patient, years		2.9 (4.9)
Meeting PA guidelines within the last 6 months		
No	340	81.7%
Yes	76	18.3%

*SD* Standard deviation, *BMI* Body mass index, *PA* Physical activity

### Prevalence and associated factors of PA advice

**Fig. 2:**
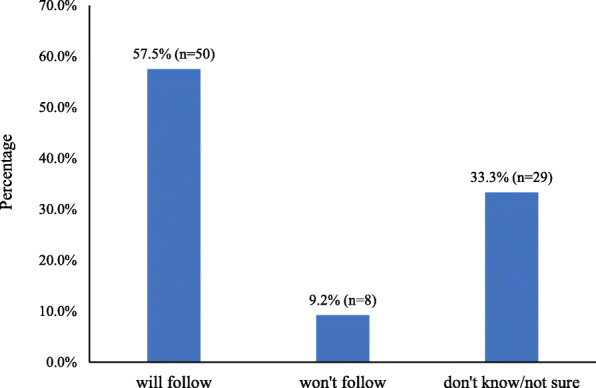
Likelihood of patients (*n* = 69) follow physical activity advice from their physicians

The prevalence of receiving PA advice was 19.2% (*n* = 87 out of 454). Less than one-fifth of patients received PA advice during physician visits. Among the 87 patients who received PA advice, 50 (57.5%) of them reflected that they would follow the physician’s PA advice, whereas only 8 (9.2%) of them reflected that they wouldn’t follow. The rest (*n* = 29, i.e. 33.3%) were either “don’t know” or “not sure” (Fig. 2).

In the univariate analysis, participants who were unemployed [odds ratio (OR): 0.29; 95% confidence interval (CI): 0.10–0.84], had longer time as a patient (OR: 0.50; 95% CI: 0.28–0.88), and had adequate PA in the last 6 months (OR: 0.30; 95% CI:0.13–0.73) were less likely to receive PA advice from their physicians (*p* < 0.05) (Table 2). In the logistic regression model, male patients were more likely to receive PA advice (OR: 1.81; 95% CI: 1.08–3.05), whereas unemployed patients (OR: 0.16; 95% CI: 0.04–0.67) and who had adequate PA in the last 6 months (OR: 0.29; 95% CI: 0.12–0.71) were less likely to receive PA advice from their physicians (Table 2).

**Table 2:** Sociodemographic and health-related factors associated with receiving PA advice from physicians

Characteristics	Unadjusted OR	95% CI	*P*-value	Adjusted OR	95% CI	*P*-value
Gender						
Female	1.00	Reference		1.00	Reference	0.03*
Male	1.47	0.92–2.35	0.11	1.81	1.08–3.05	
Age					Not retained^a^	
<47	1.00	Reference				
≥ 47	0.80	0.48–1.33	0.39			
Education					Not retained	
Less than Secondary school	1.00	Reference				
Secondary school	1.23	0.65–2.34	0.52			
More than secondary school	0.74	0.39–1.40	0.35			
Employment status						
Employed	1.00	Reference		1.00	Reference	
Unemployed	0.29	0.10–0.84	0.02*	0.16	0.04–0.67	0.01*
Retired	1.32	0.73–2.39	0.36	1.34	0.72–2.50	0.36
BMI					Not retained	
Underweight	1.00	Reference				
Normal weight	1.03	0.33–3.21	0.96			
Overweight	0.78	0.23–2.68	0.69			
Obese	1.39	0.43–4.43	0.58			
No. of years as a patient					Not retained	
< 2.9	1.00	Reference				
≥ 2.9	0.50	0.28–0.88	0.02*			
Meeting PA guidelines within the last 6 months						
No	1.00	Reference		1.00	Reference	
Yes	0.30	0.13–0.73	0.01*	0.29	0.12–0.71	0.01*

*OR* Odds ratio*, CI* Confidence interval, *BMI* Body mass index, *PA*, physical activity

^a^These factors were not included in the multivariate model

*p-value < 0.05

### Details of PA advice

Among those who received PA advice, very few instances of PA advice (9.2%, *n* = 8) provided by physicians contained all of the important elements of PA prescription (i.e., frequency, intensity, duration and type). Although the type of exercise was most frequently specified (55.2%, *n* = 48), most PA advice was missing either frequency, intensity or duration (Table 3). When details on frequency, intensity or duration were given, most included suboptimal intensity (i.e., low intensity) and duration (i.e., < 30 min per session). Aerobic activities were most frequently recommended than other types of activity (Table 3).

**Table 3:** Type of physical activity advice provided by physicians (*N* = 87)

Variable	n	Percentage
Frequency	19	21.8%
< 5 sessions per week	5	26.3%
≥5 sessions per week	14	73.7%
Intensity	28	32.2%
Low intensity	21	75.0%
Moderate to Vigorous intensity	7	25.0%
Duration	20	23.0%
<30 min per session	6	30.0%
≥30 min per session	14	70.0%
Type	48	55.2%
Walking	12	25.0%
Swimming	12	25.0%
Stretching exercise	9	18.8%
Badminton	7	14.6%
Muscle-strengthening activities	6	12.5%
Mind-body exercise	4	8.3%
Running	3	6.3%
Square dancing	1	2.1%
Frequency*Intensity*Duration*Type	8	9.2%

Of the 87 patients who received PA advice, only two patients (2.3%) received comprehensive recommendations in accordance with the WHO aerobic PA and muscle-strengthening PA guidelines (Table 4).

**Table 4:** Number of components of WHO aerobic PA guidelines provided by physicians

Number of components	% of patients (*n* = 87)	Definition
4	2 (2.3%)	In line with the aerobic PA guideline^a^
3	5 (5.7%)	Partially in line with the aerobic PA guideline^b^
2	8 (9.2%)	
1	27 (31.0%)	
0	45 (51.7%)	Not in line with the aerobic PA guideline^c^

*PA* Physical activity

^a^Patients reported receiving all four components (frequency, intensity, duration, and type) as outlined in the WHO aerobic PA guidelines (WHO, 2020b)

^b^Patients reported receiving only three, two or one element outlined in the WHO aerobic PA guidelines

^c^Patients reported receiving none of the four elements outlined in the WHO PA aerobic guidelines

## Discussion

The present study investigated the prevalence of PA advice received by patients with chronic diseases, and key factors associated with the provision of this advice. To address limitations in previous studies, we also report on the content of the PA advice being given by physicians, which, to our knowledge, has not been previously examined in China.

A main finding of our study is that less than one-fifth of the patients (19.2%) reported receiving PA advice from their physician. Among them, only two patients received full-details of PA advice from their physicians. This prevalence of PA advice is lower than that reported in other countries, where a total of 31.0%, 32.8%, and 57.7% of patients reported receiving PA advice in United Kingdom (UK), Germany and U.S., respectively [17, 36, 37]. The heterogeneous questions used in these studies to quantify the level of PA advice may be responsible for these varying outcomes. In several studies, participants were commonly asked ‘has a *healthcare provider* advised you to exercise within the past 12 months’ [11, 38, 39]; whereas in the current study, patients were asked whether they were advised to exercise by their *physicians*. It is possible that other healthcare providers (e.g., nurses) may have provided our patients with PA advice during their hospital visits. Moreover, the low prevalence of giving PA advice may reflect the fact that exercise prescription in clinical setting has not yet receive much attention in China. Unlike the Green Prescription (GRx) project of New Zealand and the National Physical Activity Plan of U.S. [40, 41], which were well-promoted since 1998 and 2007, respectively, the importance of exercise advice in clinical setting was recently being emphasized in the “Healthy China 2030 Plan” [42].

Perhaps one encouraging result from this study, was that nearly 60% of respondents (57.5%, *n* = 50/87, Fig. 2) indicated willingness to follow the physician’s PA advice if provided, versus only 9.2% (*n* = 8/87) indicated not willing to follow physician’s PA advice. Such a large gap suggested that physician’s PA advice is a powerful channel to motivate PA participation among patients. Similar to previous ACSM survey reported that 65% of American who visited doctors indicated willingness to follow PA advice [43], which was considered much higher than most other exercise promotion campaigns. However, the finding need to be interpreted with cautious, as there may be social desirability bias. It is unfortunate that only 19.2% of the physicians in China prescribed PA advice, there may be a high chance of success (i.e. increase PA practice) if more physicians in China learned and are willing to prescribe PA to their patients.

The present study revealed that patients who were male were more likely to receive PA advice from their physician. This finding is consistent with previous studies involving adults of different ages [17, 30, 44]. It was reported that the prevalence of mortality from chronic diseases and obesity in Chinese men was much higher than women, it is logical that physicians in China tended to provide more suggestions, such as PA advice, to men more than women [45, 46]. Another large scale PA survey on Chinese community age 35–74 years revealed that the prevalence of leisure time PA in Chinese men, both from rural and urban areas, were much higher than women (OR = 1.16 to 2.26, *p* < .001 to .05, across various age groups) [47], hence physicians may have stronger confidence and motivation to prescribe PA for male patients due to the higher readiness in male patients.

Association between employment status and receiving PA advice was not found in American patients [18, 21, 48]. In contrast, unemployed patients in the current study were less likely to receive PA advice from their physicians. Reason for the impact of employment status in Chinese patients on receiving PA advice is unclear, however, it may be related to the income status. A recent review suggests that higher income is likely to induce Chinese individuals to utilize healthcare services including access to PA facilities (e.g., health club membership) [49], unemployed Chinese may have limited resource to access PA due to lower social economic status (SES). Physicians may therefore hesitate to provide PA advice to patients with lower SES. Further study is needed to verify the association between SES and physician’s advice on PA.

Lastly, the present study revealed that meeting PA guidelines within the last 6 months was negatively associated with receiving PA advice. This result is consistent with several other studies, that suggest sedentary patients are more likely to receive PA advice [38, 50, 51]. These findings suggest that physicians are promoting PA to patients who may benefit most from the recommendation [37, 52]. Conversely, in Brazil, patients reported having leisure-time PA were more likely to receive PA advice [53, 54]. Similarly, patients with higher PA levels tend to initiate more conversations with their providers about PA compared to those with poorer lifestyle habits [55].

We found that only 9.2% patients reported receiving complete PA advice (including frequency, intensity, duration and type) from their physicians. The lack of completed PA guidance may suggest that Chinese physicians do not receive sufficient training on exercise prescription. Previous studies suggest that healthcare providers may feel uncomfortable or lack confidence in giving specific rather than general advice [56, 57]. Other barriers to providing specific advice may include limited consultations time, lacking resources, or negative attitude toward the effectiveness of PA promotion [16]. Given the greater effectiveness of detailed PA recommendations, frequency, intensity, duration, and type should be incorporated into PA promotion practices [58].

Findings from the present study showed that a majority of patients (25.0%) were advised to participate in walking, which is generally consistent with previous studies [22, 29]. This finding may be related to the time constraints of the physicians. In China, physicians typically see up to 40 patients per day, necessitating that PA advice be quick and easily understood [59]. Instruction on walking can be given fairly quickly without much explanation in a busy clinical setting [21]. Besides walking, physicians should also learn other effective types of physical activities for patients such as resistance training and aquatic exercise.

This is the first study to investigate the prevalence of PA advice provision for patients with chronic diseases in China. The receipt of PA advice was captured at the completion of the clinic visit, reducing the risk of recall bias. Furthermore, the individual components of the PA advice were examined in this study, providing a glimpse into the type and quality of PA advice being provided to patients by their physicians.

However, there are some limitations with this study that deserve mention. The results of the study are based on self-reported data, which may lead to an overestimation of the prevalence rates of PA advice due to social desirability bias. Additionally, there may be other factors, not captured by this survey, that influence the receipt of PA advice, such as characteristics of the healthcare providers or the self-rated health status of the patients [30]. Only the provision of physicians’ advice collected from the out-patient clinics of the three major hospitals in Shenzhen China were involved, the total number of physicians involved was not known and was estimated at around 76 physicians according to the hospitals’ website. Cautious should be made as results we discussed only applied to these out-patient physicians. Another limitation is that the medical condition (i.e., acute or severe health issues) of the patients may make PA advice inappropriate, leading to an underestimation of correctly provided PA advice. Further, only three general hospitals in Shenzhen were selected may limit the external validity of our results. However, the included hospitals were major hospitals that provide services to majority of patients with various health conditions. The health services provided were similar to other general hospitals in Shenzhen, China. It is important to note that the objective of the study was to explore the provision of PA advice given by physicians from the perspective of patients, the actual practice and attitude of providing PA advice from the physician’s perspective is unknown and separate investigation is needed.

## Conclusions

Only a small proportion of patients are receiving PA advice and guidance from their physicians in healthcare settings in Shenzhen, China. In patients who received PA advice, frequency, intensity, duration, and type of activity were not commonly provided. Although patients who are most likely to benefit from PA reported receiving advice at a greater rate, there is still much room for improvement [18]. Comprehensive initiatives, such as EIM, can provide reference and guidance to improve the PA counselling practices of physicians in healthcare settings.

## Supplementary Information

**Additional file 1.** Provision of Physical Activity Advice for Patients with Chronic Diseases. Additional file 1 is the questionnaire that we developed and used for data collection.

## Data Availability

The datasets used and analyzed during the current study are available from the corresponding author on reasonable request.

## Abbreviations

ACSM: American College of Sports Medicine
AMA: American Medical Association
BMI: Body mass index
BRFSS: Behavioral Risk Factors Surveillance System
CI: Confidence interval
COPD: Chronic obstructive pulmonary disorder
EIM: Exercise is Medicine
GRx: Green Prescription
HIV: Human immunodeficiency virus
MVPA: Moderate to vigorous intensity PA
OR: Odds ratio
PA: Physical activity
SD: Standard deviation
SES: Social economic status
UK: United Kingdom
U.S.: United States
WHO: World Health Organization
