# Supplementary material for: Provision of physical activity advice for patients with chronic diseases in Shenzhen, China
Source: BMC Public Health. 2021 Nov 23;21:2143. doi: 10.1186/s12889-021-12185-7 (PMC8611940; doi:10.1186/s12889-021-12185-7)
Supplement: Supplementary file 1 — Additional file 1. Provision of Physical Activity Advice for Patients with Chronic Diseases. Additional file 1 is the questionnaire that we developed and used for data collection. [file 12889_2021_12185_MOESM1_ESM.pdf]

Provision of Physical Activity Advice for Patients with Chronic Diseases

PART I

1. What is your primary purpose for your visit to physician?

☐ Alzheimer's
☐ Asthma
☐ Osteoporosis
☐ Depression or Anxiety
☐ Low Back Pain
☐ Hypertension
☐ Blood Lipid Disorders
☐ Pre-Diabetes
☐ Osteoarthritis
☐ Inflammatory Bowel Disease
☐ Diabetes
☐ Parkinson's Disease
☐ Rheumatoid Arthritis
☐ Chronic Liver Disease
☐ Chronic Kidney Disease
☐ Fibromyalgia
☐ Peripheral Arterial Disease
☐ Chronic Obstructive Pulmonary Disorder
☐ Cancer (Colorectal Cancer, Prostate Cancer, Breast Cancer)
☐ Heart Failure
☐ Others ___________

2. How long have you became a patient of this hospital? ___________

3. How long was the consultation time in this visit? ___________

4. What did the physician prescribe just now?

4.1. Medication
☐ Yes ☐ No
4.2. Nutrition advice
☐ Yes ☐ No
4.3. Psychological advice
☐ Yes ☐ No
4.4. Physical activity advice
☐ Yes (If "Yes", please go to question 5) ☐ No (If "No", please go to question 7)

5. What are the contents of PA advice provided by physician?

5.1. Physical activity frequency
☐ ___________ times/week ☐ Not specified
5.2. Physical activity intensity
☐ Low intensity ☐ Moderate intensity
☐ High intensity ☐ Not specified
5.3. Physical activity duration
☐ ___________ minutes/session ☐ Not specified
5.4. Physical activity type
☐ ___________ ☐ Not specified

6. Will you follow the physical activity advice given by physician (If "No", please specify the reason)?

☐ Yes ☐ No ___________ (reason)
☐ Not sure/I don't know

Copyright © protected. Please do not use nor quote without the permission from authors.

7. During the last 6 months, did you participate in any physical activity? (If “Yes”, please go to question 8; If “No”, please go to “PART II”)
   ☐ Yes (go to question 8) ☐ No (go to PART II)

8. What are the contents of your PA?
   8.1. Physical activity intensity
      ☐ I only participate in low intensity PA (Please go to “to “PART II”)
      ☐ I participate in moderate-to-vigorous PA
   8.2. Physical activity frequency ___________ times/week
   8.3. Physical activity duration ___________ minutes/session
   8.4. Physical activity type ___________

PART II

Gender: ☐ Male ☐ Female Height: ________ cm Weight: ________ kg

Age: ___________ years or ☐ 18-25 years ☐ 26-30 years
☐ 31-35 years ☐ 36-40 years
☐ 41-45 years ☐ 46-50 years
☐ 51-55 years ☐ 56-60 years
☐ 61-65 years ☐ 66 years and above

Employment status:
☐ Unemployed ☐ Employed ☐ Self-employed
☐ Retired ☐ Others

Household Monthly Income:
☐ 0-5,000 RMB ☐ 5,001-10,000 RMB
☐ 10,001-15,000 RMB ☐ 15,001-20,000 RMB
☐ 20,001-25,000 RMB ☐ 25,001-30,000 RMB
☐ 30,001-35,000 RMB ☐ 35,001-40,000 RMB
☐ 40,001- 45,000 RMB ☐ 45,001-50,000 RMB
☐ > 50,000 RMB

Marital Status:
☐ Married ☐ Single ☐ Others

Education Background:
☐ Uneducated/Pre-primary Education
☐ Primary Education
☐ Secondary Education
☐ Higher Education (Junior College)
☐ University Graduate (Bachelor)
☐ Advanced Education (Master and above)
☐ Others ___________

Copyright © protected. Please do not use nor quote without the permission from authors.
